# Analysis of Changes in Selected Physicochemical Parameters and Elemental Composition of Honey as a Result of Adulteration with Sugar Additives

**DOI:** 10.3390/foods15030562

**Published:** 2026-02-05

**Authors:** Magdalena Gajek, Karolina Moj, Piotr Wysocki, Elżbieta Kuśmierek, Małgorzata Iwona Szynkowska-Jóźwik

**Affiliations:** Institute of General and Ecological Chemistry, Faculty of Chemistry, Lodz University of Technology, Zeromskiego 116, 90-924 Lodz, Poland; 237401@edu.p.lodz.pl (K.M.); piotr.wysocki@p.lodz.pl (P.W.); elzbieta.kusmierek@p.lodz.pl (E.K.); malgorzata.szynkowska@p.lodz.pl (M.I.S.-J.)

**Keywords:** honey adulteration, elemental analysis, physicochemical parameters, sugar syrups

## Abstract

Honey authenticity is increasingly threatened by the addition of low-cost sugar syrups and substitutes, which reduce its nutritional value and market credibility. In this study, five types of Polish honeys (honeydew, forest, multifloral, nectar–honeydew, and rapeseed) were intentionally adulterated with beet syrup, beet molasses, invert syrup and artificial honey at levels of 10% and 50% (*v*/*v*). The impact of adulteration was evaluated using elemental profiling by ICP-OES combined with physicochemical analyses (water content, sugar content and electrical conductivity) and chemometric methods (PCA and HCA). Natural honeys were characterized by high K, Mg and Ca contents and low Na levels, whereas adulterants significantly altered mineral composition, leading to a marked decrease in key authenticity ratios, particularly K/Na (decreases exceeding 90% at the 50% adulteration level, with systematic shifts already observable at 10% addition). Beet molasses caused the strongest disturbances in macroelement balance, while invert syrup induced weaker effects. Adulteration also resulted in increased water content, reduced °Brix values and pronounced changes in electrical conductivity. Chemometric analysis enabled clear discrimination between natural, adulterated and sugar-based samples. The combined use of elemental ratios, physicochemical parameters and chemometrics provides a robust and sensitive approach for detecting honey adulteration and supporting authenticity control.

## 1. Introduction

The chemical composition of honey is largely influenced by its botanical and geographical origin [1]. Natural, mature honey is a dense, hygroscopic substance that can be either liquid or crystallized. The primary constituents of honey are sugars, which account for approximately 95% of its dry matter [2]. Among these, glucose and fructose are predominant, while smaller amounts of disaccharides (e.g., sucrose) and trisaccharides (e.g., melezitose) are also present [2,3]. Carbohydrates are primarily responsible for determining key physicochemical properties such as viscosity, granulation, and hygroscopicity. Proteins (e.g., albumins and globulins), enzymes (e.g., invertase, diastase, and glucose oxidase), and amino acids occur in minor amounts [2]. These compounds originate from nectar, as well as from the secretions of the pharyngeal glands and saliva of honey bees. Organic acids are present in the smallest quantities (approximately 0.5%) and include lactic, citric, butyric, propionic, formic, acetic, tartaric, succinic, oxalic, gluconic, and malic acids [1]. In addition, honey contains macro- and microelements. The mineral content ranges from 0.04% to 0.2%, and the water content of honey typically varies between 10% and 20%. The presence of macro- and microelements in honey reflects both natural processes occurring in soil and plants and potential anthropogenic contamination [2,4]. For this reason, elemental profiling has become an essential tool in quality control procedures and in investigations aimed at determining the botanical and geographical origin of honey [1,2]. It is also worth noting that certain elements may serve as varietal chemical markers. For instance, buckwheat honey is typically characterized by a higher manganese content, while linden and acacia honeys tend to exhibit elevated levels of potassium or calcium. Such elemental profiles can be valuable in the authentication of honey varieties, particularly in the context of regional products or those protected by geographical indications [1].

According to the Codex Alimentarius Standard for Honey (Codex Stan 12-1981, amended 2022), an international food standard developed by FAO and WHO and referenced by the European Union, honey intended for human consumption in any form must be free from both organic and inorganic substances that do not occur naturally in honey, and must retain its nutritional and health-promoting properties to ensure that it does not negatively affect either product quality or human health [5]. Moreover, honey intended for human consumption must meet defined physicochemical quality criteria specified in the aforementioned Codex. These include limits for moisture (≤20%, or ≤23% for heather honey), minimum levels of fructose plus glucose (≥60 g/100 g for nectar honeys; ≥45 g/100 g for honeydew honeys), and maximum sucrose content (generally ≤5 g/100 g, with higher limits permitted for specific botanical origins). Additional quality parameters include water-insoluble solids (≤0.1 g/100 g), free acidity (≤50 meq/kg), minimum diastase activity (≥8 Schade units), and hydroxymethylfurfural content (≤40 mg/kg; ≤80 mg/kg for tropical honeys), supporting both product quality and consumer protection. The Codex Alimentarius Standard for Honey also defines limits for electrical conductivity, which is used as a classification parameter reflecting the mineral and organic acid content of honey. For most nectar honeys, the electrical conductivity should not exceed 0.8 mS/cm, whereas higher values (≥0.8 mS/cm) are characteristic of honeydew honeys and certain botanical varieties, such as chestnut honey. This parameter therefore supports the differentiation of honey types and the detection of compositional anomalies [6].

**Figure 1:**
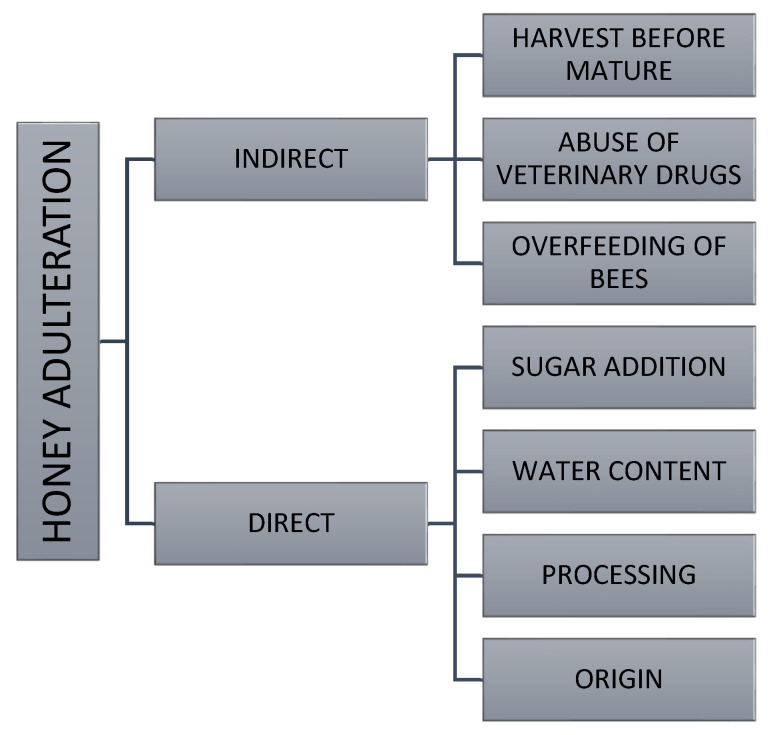
Possible categories of honey adulteration.

One of the most common methods of honey adulteration at the production stage is the direct addition of cheaper sugar syrups—such as those derived from beet, corn, cane, or invert sugar, as well as glucose–fructose syrups or syrups with a high sucrose content—which has been widely reported in the literature as a major challenge for honey authenticity control [3,4,7,8,9]. Such modifications have an impact not only on the quality of the product but also the beekeeping sector as a whole, disrupting the market, damaging sales, and undermining consumer trust in honey. These practices are largely economically motivated, as the substantial price disparity between authentic honey and low-cost sugar syrups makes adulteration financially attractive, leading to unfair competition and significant economic losses within the honey market [10,11]. Another issue is the excessive feeding of bees with the aforementioned syrups, which also adversely affects the nutritional value of honey [10,12]. Water is one of the key components of honey and plays a critical role in determining its shelf life. The water content in honey depends on several factors, including storage conditions, harvest timing, and the prevailing climate. These factors may influence important physical characteristics such as crystallization and viscosity. Premature harvesting often results in higher water content than recommended, accelerating fermentation processes and negatively impacting both the product’s stability and its commercial appeal [13,14]. Many consumers still erroneously believe that natural honey should not crystallize and tend to prefer its liquid form. In response, some producers resort to uncontrolled heating to improve product appearance and boost sales. This process removes air bubbles and liquefies glucose microcrystals, but it also degrades honey quality, reduces water content, and leads to the formation of hydroxymethylfurfural (HMF) [10,12]. Figure 1 presents an overview of honey adulteration practices, categorized into direct and indirect methods.

To assess the originality and origin of honey, elemental composition analysis is commonly employed, with inductively coupled plasma-based techniques (ICP-OES and ICP-MS) being among the most widely used approaches. Numerous studies have demonstrated the use of ICP-based methods for honey characterization, particularly for botanical and geographical origin determination, safety assessment, and method development, including investigations on honeys from different geographical regions worldwide, such as Europe and Poland (Table S1 in Supplementary Materials) [15,16,17,18,19,20,21,22]. The elemental composition of honey varies according to botanical origin and can therefore serve as a potential authenticity marker [23]. In this context, ICP-OES represents a robust and accessible analytical technique that may serve as a cost-effective screening tool for detecting compositional changes associated with honey adulteration. One of the first approaches applied in authenticity testing was the analysis of the 13C/12C isotope ratio (SCIRA), effective in detecting syrups derived from C4 plants, but less useful for C3 sugars. The comparison of isotope ratios in proteins and sugars allows the estimation of adulteration [10,23]. Chromatographic methods such as TLC, HPLC, GC, LC, HPAEC-PAD, and GC-MS are increasingly employed [10,12,24]. GC enables detection of HFCS addition at a 5% level (marker–anhydrodifructose) [25]. HPLC is applied to determine HMF content, monitor oligosaccharides (HPLC-RID), and identify other adulteration markers [10,24]. UHPLC-Q-TOF-MS and GC-MS have also been used to detect indirect adulteration resulting from bee feeding with sugar syrups [24,25,26]. Spectroscopic techniques (NIR, MIR, ATR-FTIR, Raman) have also gained wide application [10,25,26]. NIR is rapid, non-destructive, and cost-effective, whereas Raman spectroscopy is less affected by water molecules. Adulteration with syrups (e.g., maltose, HFCS) produces characteristic spectral shifts [10,27,28]. NMR, despite its high cost, provides precise structural information [10,29]. Novel approaches include the electronic tongue, which analyzes compounds influencing honey’s organoleptic properties, as well as the study of exogenous DNA and miRNA from adulterating sugars. However, these methods are expensive and time-consuming [30,31]. Chemometric tools (PCA, PLSR, PLS-LDA, OPLS-DA) remain essential for reducing sample complexity and classifying honeys based on similarity [12,24].

The objective of this study was to evaluate the authenticity of honeys by integrating ICP-OES-based elemental composition profiling with physicochemical analysis. Pure honeys of different botanical origins, common sugar-based adulterants, and their mixtures were examined to investigate systematic compositional changes associated with adulteration and to assess the potential of ICP-OES as a practical and cost-effective screening tool for honey authenticity. The study focused in particular on macroelement ratios and complementary parameters such as electrical conductivity, water content, and sugar content [32].

## 2. Materials and Methods

### 2.1. Samples

Five types of natural honey into which selected sugar-based products were intentionally introduced at levels of 10% and 50% (*v*/*v*) to simulate adulteration were used in the study. The additives included artificial honey and inverted syrup (synthetic adulterants), as well as concentrated beet syrup and beet molasses (natural adulterants). The concentrations and types of adulterants were chosen based on literature data as the most commonly reported ‘fillers’ in natural honey [24,33,34]. In addition, artificial honey was included as a reference product, representing a low-cost substitute for natural honey. The natural honey samples represented different botanical types, including nectar honeys, i.e., honeys produced predominantly from floral nectar, honeydew honeys derived mainly from plant secretions or excretions of plant-sucking insects, and mixed nectar–honeydew honeys. The selection of honey samples was intentionally designed to cover a broad range of compositional and mineral characteristics commonly observed in commercial honeys. Monofloral nectar honeys were included as matrices typically characterized by lower mineral content, whereas honeydew and mixed nectar–honeydew honeys were selected due to their generally higher mineralization and distinct elemental profiles. This approach enabled the assessment of elemental trends across contrasting honey matrices and supported the evaluation of the applicability of ICP-based elemental analysis as a screening tool for honey authenticity. All honey samples originated from the 2024 harvest season, with collection periods corresponding to the natural harvesting times of individual honey types. The examined materials used in the study integrally come from Poland. To obtain the mixtures, appropriate volumes of honey and each additive were homogenized until a uniform consistency was achieved. Each of the five honey samples was mixed individually with each additive at two concentration levels (10% and 50%, *v*/*v*), resulting in a total of 49 analytical samples prepared for analysis. Detailed information about samples is summarized in Table 1.

### 2.2. Chemicals and Reagents

Nitric acid (65%, Baker, Avantor Performance Materials Poland S.A., Gliwice, Poland) was used for sample mineralization. Calibration solutions were prepared using a multi-element ICP standard solution (ICP Multi-Element Standard, CAPchem, Bogomilovo, Bulgaria; 1000 mg/L). Additional single-element standard solutions were used for sulfur (Merck, Darmstadt, Germany), phosphorus (Radian International LLC, Austin, TX, USA), and ytterbium (SCP Science, Baie-d’Urfé, QC, Canada). Method accuracy was verified using TMDA-64 (fortified lake water sample, National Water Research Institute, Burlington, Halton, ON, Canada) and NCS ZC 8100 2b (human hair, China National Analysis Center for Iron & Steel and NCS Testing Technology Co., Ltd., Beijing, China).

### 2.3. Research Procedure

#### 2.3.1. Elemental Analysis

For elemental analysis, all materials (pure honeys, additives, and their mixtures) were subjected to mineralization under identical conditions. An aliquot of approximately 0.5 g of each sample was accurately weighed using an analytical balance (with a readability of 0.00001 g, Pioneer Ohaus^®^, Nänikon, Switzerland) into a digestion vessel, followed by the addition of 65% nitric acid. The mineralization process was performed in a closed-vessel microwave system (Ultrawave, Milestone, Sorisole, Italy) using a program optimized for organic matrices. The resulting digests were quantitatively transferred into 50 mL volumetric flasks, spiked with an ytterbium internal standard (10 mg/L), and diluted to volume with deionized water. Procedural (reagent) blank samples were prepared in the same way as the studied samples. The procedure of mineralization was analogous to that described previously for the bee products in the study by [15].

Elemental concentrations were quantified using the ICP-OES technique (Thermo Scientific (Waltham, MA, USA), iCAP 7000 series). For major elements such as Ca, K, Mg, and Na, measurements were carried out in the radial plasma view, whereas the remaining analytes (Ag, Al, Ba, Cd, Co, Cr, Cu, Fe, Mn, Mo, Ni, P, Pb, S, Sb, Sn, Sr, Ti, V, and Zn) were determined in the axial mode. Each type of sample was digested once, and instrumental measurements were performed in triplicate. Ytterbium, added as an internal standard, was employed to control signal stability during the measurements. Detailed instrumental parameters used in the multi-element determinations are summarized in Table 2.

Calibration curves were prepared from a multi-element standard solution of CAPchem with an initial concentration of 1000 mg/L, while additional single-element standards were used for S, P, and Yb. All standards were diluted to match the sample matrix. Measurements of standards and samples were carried out within a single analytical session.

As no certified material with a honey-like matrix was available, accuracy was verified using two reference materials—TMDA-64 (fortified lake water sample) and NCS ZC 8100 2b (human hair)—which together covered the target elements. The limits of detection (LOD) and quantification (LOQ) demonstrated adequate sensitivity for both macro- and trace elements. For macroelements (K, Na, Ca, Mg, P and S), LOD values ranged from 0.003 to 0.05 mg/kg, while LOQ values were between 0.009 and 0.15 mg/kg. For the remaining trace elements analyzed, LOD values ranged from 0.3 µg/kg to 24 µg/kg, and LOQ values from 1 µg/kg to 71 µg/kg. Detailed validation parameters, along with the obtained values for the analyzed certified reference materials, are provided in Table S3 (Supplementary Materials).

#### 2.3.2. Determination of Water Content

In order to determine the water content of the selected honey samples, analyses were performed at room temperature using a laboratory refractometer (Carlzeiss Jena 197338, Jena, Germany). A few drops of honey (or other tested product) were placed on the dry prism with a metal laboratory spoon, evenly spread, and covered with the second prism. The instrument was properly adjusted, the refractive index was read from the scale, and the water content (percentage by weight) was determined using the tables provided in the Regulation of the Minister of Agriculture and Rural Development of 14 January 2009, on analytical methods for honey evaluation [32].

#### 2.3.3. Determination of Sugar Content

To determine the sugar content of the selected bee products and their additives, refractometric measurements were carried out at room temperature using a portable refractometer commonly applied in beekeeping (ATC). A few drops of the sample were placed on a dry prism, evenly spread, covered with the second prism, and measured. The sugar content was then directly read from the instrument scale. The measurements were carried out in accordance with the Polish Regulation of the Minister of Agriculture and Rural Development of 14 January 2009 on methods of analysis related to the assessment of honey quality [32], and the results were expressed as °Brix.

#### 2.3.4. Determination of Electrical Conductivity

A sample of approximately 20 g of honey was weighed in terms of dry matter, which was calculated according to the following formula:

$$M=\frac{20\;g\;\cdot 100}{MS}$$

where:

*M*—required weight [g];
*MS*—dry matter content, which is 100% minus water content.

A weighed honey sample was dissolved in demineralized water in a 100 mL volumetric flask and filled to the mark. The solution was transferred into a beaker, and the conductivity cell was immersed to perform the measurement. The electrical conductivity (EC) of honey was then calculated from the measured value using the following formula:

$$S_{H}=K\cdot G$$

where:

*S_H_*—electrical conductivity of the honey solution expressed in mS·cm^−1^;
*K*—cell constant expressed in cm^−1^;
*G*—conductivity expressed in mS.

The electrical conductivity was measured using CPC-401 pH/conductivity meter (Elmetron, Zabrze-Grzybowice, Poland) and expressed in mS.

EC was determined in accordance with the Polish Regulation of the Minister of Agriculture and Rural Development of 14 January 2009 on methods of analysis related to the assessment of honey quality [32].

#### 2.3.5. Statistics and Chemometrics Analysis

Statistical analysis was performed using STATISTICA 12.5 and R 4.5.0. The normality of data distribution was verified using the Shapiro–Wilk test. As the distributions of most parameters deviated from normality (*p* < 0.05), non-parametric statistical tests were applied. Differences between natural honeys, sugar-based adulterants and adulterated samples were evaluated using the Kruskal–Wallis test at a significance level of *p* < 0.05. When the Kruskal–Wallis test indicated statistically significant differences, post hoc pairwise comparisons were performed using Dunn’s test with adjustment for multiple comparisons.

For multivariate data exploration and pattern recognition, principal component analysis (PCA) was applied to the combined dataset of elemental concentrations and physicochemical parameters. Hierarchical cluster analysis (HCA) was performed using Ward’s method with Euclidean distance as a measure of similarity. Prior to PCA and HCA, the data were standardized using z-score normalization. Chemometric analyses were used to visualize similarities and differences between pure honeys, adulterants and their mixtures and to evaluate the effect of adulteration on the overall composition of honey.

## 3. Results and Discussion

### 3.1. Elemental Analysis

In this study, 24 elements were analyzed in a total of 49 bee product samples. Their concentrations (Ag, Al, Ba, Ca, Cd, Co, Cr, Cu, Fe, Mn, K, Mg, Mo, Na, Ni, P, Pb, S, Sb, Sn, Sr, Ti, V, and Zn) were determined using ICP-OES. The selection of elements was guided by their relevance to (i) honey authenticity and botanical origin, and (ii) consumer safety. Macroelements (K, Na, Ca, Mg, together with P and S) were prioritized because they constitute the main mineral fraction of honey and their profile reflects both botanical origin and environmental conditions [35]. They also respond in a predictable manner to dilution with sugar syrups, making derived ratios (e.g., K/Na) informative authenticity markers [36,37]. In addition, trace elements (e.g., Mn, Fe, Cu, Zn, Sr, Al, Ti) were included to capture complementary environmental and botanical fingerprints that support multivariate discrimination between pure and adulterated samples. Finally, potentially toxic elements (Ag, Ba, Cd, Cr, Mo, Pb, Sb, Sn, and V) were monitored as safety indicators; in the present dataset, these were mostly below detection limits, suggesting no measurable contamination in the analyzed samples (Table S2, Supplementary Materials).

Among the examined macroelements, potassium was the dominant constituent of natural honeys. In pure samples, potassium concentrations ranged from approximately 200 mg/kg in nectar honeys to over 3600 mg/kg in honeydew and forest honeys (Table S2). Honeydew and forest honeys showed comparable levels of other macroelements, with calcium, magnesium, sodium, phosphorus, and sulphur concentrations of approximately 35, 88, 30, 150, and 115 mg/kg, respectively. In contrast, nectar–honeydew honeys were characterized by the highest sodium and calcium contents within the analyzed samples, reaching 271.1 mg/kg for Na and 60.78 mg/kg for Ca. Among the tested adulterants, molasses exhibited the highest concentrations of all investigated macroelements. In particular, the contents of calcium, potassium, magnesium, sodium, phosphorus, and sulphur reached 895.5, 305 969, 38.63, 9461, 95.64, and 2026 mg/kg, respectively. Molasses was also the only analyzed adulterant in which cobalt was detected (0.801 mg/kg). Consequently, Co was observed in honey–molasses mixtures, but only at the 50% adulteration level, with concentrations ranging from 0.448 mg/kg in multifloral honey to 0.697 mg/kg in forest honey. The addition of sugar-based adulterants caused pronounced and systematic changes in the elemental composition of honeys. Overall, two distinct patterns were observed depending on the adulterant used: (i) low-mineral sugar syrups produced a systematic dilution of the native mineral profile of honey, whereas (ii) mineral-rich additives, such as molasses and thick beetroot syrup, increased the concentrations of selected macroelements, particularly potassium and sodium (Table S2). At the 50% adulteration level with low-mineral sugar syrups (S.I. and Sz), macroelement concentrations typically decreased by approximately 40–60%. For example, potassium decreased from 3665 to 1494 mg/kg in forest honey (L+S.I. 50%) and from 1291 to 538.3 mg/kg in nectar–honeydew honey (NS+S.I. 50%) (Table S2). In contrast, molasses or thick beetroot syrup—characterized by very high mineral content—led to an enrichment of selected macroelements, most notably potassium and sodium.

In the natural honeys analyzed in this study, the K/Na ratio was generally high, especially in honeydew (128.6) and forest (110.1) honeys, indicating a dominant presence of potassium with a relatively low sodium content (Table 3). Nectar honeys showed lower ratios, consistent with their more “diluted” mineral composition (<51). The addition of sugar-based adulterants caused pronounced shifts in these ratios. Syrups and artificial honeys were characterized by lower potassium and magnesium contents and relatively higher sodium, leading to a decrease in the K/Na ratio in the mixtures. The most prominent effect was observed for beet molasses, which, despite its high potassium content, also introduced significant amounts of sodium, thereby strongly disturbing the original balance.

Overall, the observed trends show that adulterants, regardless of type, tend to reduce the characteristic macro-element ratios of pure honeys, although the direction and magnitude of changes depend on the specific additive. As a result, adulterated honeys lose their distinctive mineral profile and the evaluation of elemental ratios provides a clear and reliable approach for authenticity assessment.

**Figure 2:**
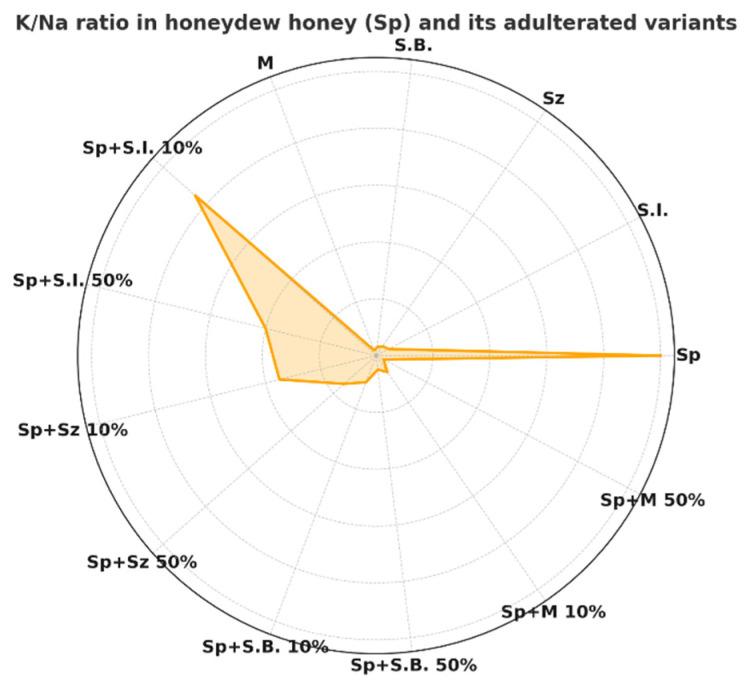
Radar plot of the K/Na ratio for honeydew honey and its mixtures with different sugar-based adulterants.

The pure honeydew honey showed the highest K/Na ratio (128.6) among all analyzed samples, reflecting its mineral-rich composition (Figure 2, Table 3). The addition of adulterants led to a marked decrease in this ratio, particularly in the case of beet molasses (97.4% decrease at the 50%), beet syrup (95.1 decrease at the 50%) and artificial honey (91% decrease at the 50%), which strongly distorted the original profile. In contrast, invert syrup caused a smaller but still substantial reduction in the K/Na ratio, with decreases of approximately 56% already observed at the 10% adulteration level. This highlights that the extent of changes in elemental ratios depends not only on the proportion but also on the type of adulterant, making such ratios a sensitive and informative tool for honey authenticity assessment. Taken together, these findings indicate that ICP-OES–based elemental profiling represents a practical and cost-effective screening approach for detecting honey adulteration, particularly when elemental ratios such as K/Na are considered, thereby addressing the need for accessible methods suitable for routine quality control.

Gręda et al. (2025) [36] evaluated the potential of elemental analysis (Na, K, Mg, Ca, Rb) for honey authentication. In their study, natural honeys of different botanical origins were compared with syrups and artificial honeys commonly used for adulteration (including invert syrups, beet syrups, and artificial honey). The authors demonstrated that natural honeys are characterized by high levels of K, Mg and Ca and low Na, whereas the syrups showed the opposite trend—particularly low concentrations of macroelements and significantly higher Na. As a result, the addition of such adulterants led to a pronounced decrease in the K/Na and K/Mg ratios, which proved to be sensitive indicators of adulteration. Importantly, even a relatively small proportion of syrup in honey was sufficient to markedly disturb these ratios, supporting the use of elemental indicators for detecting honey fraud. Similar studies were conducted by Czipa et al. (2018) [37]. Researchers analyzed 140 monofloral honey samples and determined the concentrations of six macroelements (K, Ca, Mg, Na, P, S) using ICP-OES. While the evaluation of absolute concentrations already provided high classification accuracy, the use of macroelement ratios, particularly K/Na and K/Mg, significantly improved the discrimination of botanical origin, reaching 100% correct classification in discriminant analysis. The authors emphasized that such ratios are more robust indicators than raw concentrations, which can be influenced by environmental and soil conditions. Importantly, they suggested that in floral honeys a K/Na ratio above 20 may be considered a threshold of authenticity, as lower values could indicate adulteration with sugar syrups or artificial honey.

Inter-element relationships were further examined using Pearson correlation analysis (Table S4 in Supplementary Materials). A Pearson correlation matrix was generated to evaluate linear relationships among the analyzed elements. Two dominant clusters of strongly associated variables were clearly visible. The first cluster, comprising macroelements such as K, Na, Ca, Fe, S and Sr, showed extremely strong positive correlations (r > 0.95). This highly coherent behavior indicates that these elements share a common origin connected to the bulk mineral composition of honey, which is largely determined by nectar and plant physiology. Their tight linear relationships also reflect their uniform response to sugar-based adulterants, which dilute or enhance these major nutrients in a predictable manner. This pattern reinforces the diagnostic relevance of macroelement ratios such as K/Na, which shift markedly upon adulteration. The second cluster contained several trace elements, including Cu, Mn, Mg, Zn and Ti, which also exhibited strong positive correlations (r typically > 0.75). These elements are strongly linked to the botanical and environmental signatures of natural honeys, as they originate primarily from plant uptake and local soil composition. Their correlated distribution across samples indicates that adulteration tends to dilute these environmentally derived trace elements consistently. In contrast, Al showed negative correlations with most macroelements (r ≈ −0.39 to −0.41), suggesting that its variability is driven by different factors such as soil dust or external environmental inputs rather than nectar composition.

Overall, the correlation matrix demonstrates that macroelements and trace elements provide complementary information for authenticity assessment. Macroelements respond strongly and linearly to adulteration, while trace elements preserve the environmental and botanical fingerprint of natural honeys, allowing reliable differentiation between pure and adulterated samples.

When interpreting changes in elemental composition induced by sugar adulteration, it is important to consider the inherent natural variability of honey. A key limitation of the proposed authenticity markers is the natural variability of honey composition, which can be substantial even within the same declared honey type. The elemental profile and related physicochemical properties (e.g., electrical conductivity) are primarily shaped by botanical origin and the associated nectar/honeydew sources, but they are also modulated by geographical location and local edaphic conditions (soil mineral availability and geochemical background), environmental exposure (including anthropogenic inputs), and seasonal/climatic factors affecting plant physiology and nectar/honeydew secretion [38,39,40,41,42]. In addition, beekeeping and post-harvest practices (e.g., feeding regimes, handling, processing temperature, and storage/packaging conditions) may contribute to further variability by altering moisture balance and/or introducing minor contamination pathways [43]. Consequently, while the combined use of elemental ratios (e.g., K/Na), physicochemical parameters, and chemometrics provided robust discrimination for the investigated Polish honeys and adulterants, the generalizability of specific threshold values and marker patterns may be limited when transferring the approach to other botanical varieties, regions, production systems, or harvest seasons. Broader multi-region and multi-season datasets will therefore be beneficial to refine reference ranges, quantify within-type variability, and strengthen external validation of the proposed markers.

However, this inherent variability does not preclude the existence of reproducible elemental patterns characteristic of specific honey types. Although natural changeability in elemental composition poses a limitation for the universal applicability of specific thresholds, a body of literature demonstrates that mineral profiles consistently reflect botanical and geographical origins of honey, providing a rationale for their use as authenticity markers. Several studies have shown that multi-element profiles can discriminate among honey types using chemometric tools, with elements such as K, Mg, Ca, Mn, Na and Fe contributing significantly to classification models according to floral source or region when combined with statistical analyses such as PCA and LDA [42,44]. For example, the relative abundances of macroelements (e.g., K > Ca > Mg > Na) and trace elements have been repeatedly reported as characteristic of honeys from different botanical groups, and chemometric classification models based on these profiles have achieved high accuracy in distinguishing monofloral and multifloral honeys [45]. Work by Pavlin et al. further indicates that specific elements (e.g., Mn, K, Ca) are influenced predominantly by the type of pollen and can serve as indicators of floral source, whereas others (e.g., Na, Mg, Fe) correlate more with environmental and geographical factors [44]. Taken together, these findings indicate that despite the natural compositional variability, elemental fingerprints are reproducible and informative, particularly when coupled with multivariate analysis, supporting their continued investigation as tools for honey authentication while acknowledging the need for region- and type-specific calibration.

### 3.2. Physicochemical Parameters

Physicochemical parameters were included in the present study due to their established role in honey quality assessment and authenticity control, as defined by the Codex Alimentarius Standard for Honey [5]. Parameters such as water content, electrical conductivity, and sugar composition are routinely used to evaluate honey maturity, stability, and botanical classification. Moreover, these parameters are sensitive to technological treatments and adulteration with sugar syrups, as the addition of exogenous sugars or water alters their values beyond those expected for natural honeys [40,46,47]. Therefore, the combined evaluation of physicochemical parameters alongside elemental composition provides complementary information for detecting compositional anomalies and assessing honey authenticity in accordance with Codex-based quality requirements. The results of water content, sugar content, and electrical conductivity for all tested honeys, adulterants, and their mixtures are summarized in Table 4, while detailed interpretations of each parameter are provided in the following subsections.

### 3.3. Determination of Water Content

**Figure 3:**
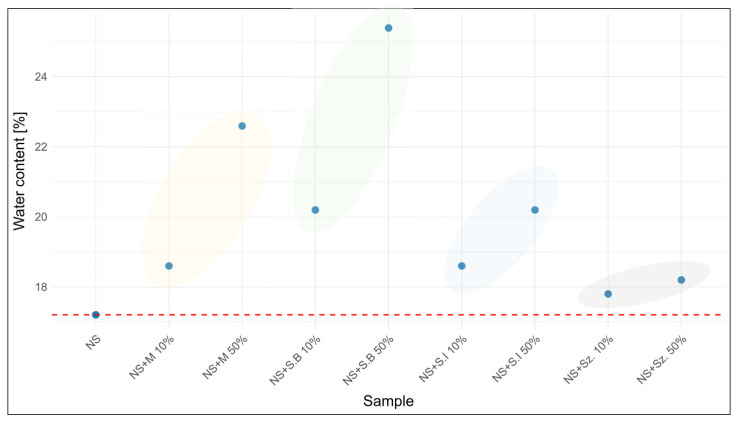
Percentage of water content in adulterated nectar-honeydew honey depending on the adulterant used and the level of adulteration.

The water content of the analyzed honeys ranged between 16.2 (forest honey) and 17.2% (nectar-honeydew honey), which is consistent with the values typically reported for unadulterated honeys and within the regulatory limit of 20% [10]. In contrast, the investigated adulterants exhibited markedly higher water contents, particularly beet syrup (>27%) and inverted syrup (22.6%), whereas artificial honey showed the lowest value among the tested additives (19.0%). As a result, the addition of syrups significantly increased the water content of honey mixtures, with the effect being more pronounced at the 50% adulteration level. In several cases (e.g., samples with beet syrup or inverted syrup), the legal threshold of 20% was exceeded, indicating that water content may serve as a useful screening parameter for detecting honey adulteration. However, the differences were less evident in the case of artificial honey, suggesting that water content alone cannot reliably detect all types of adulteration and should be complemented by additional analytical methods. As an illustration, Figure 3 presents the changes in water content for nectar–honeydew honey and its mixtures with selected adulterants. This example reflects the general trend observed across all honey types, namely an increase in water content with the addition of syrups, particularly at the 50% level. The obtained results of water content for honey samples, sugar additives, and their mixtures are presented in Table 4.

The moisture content of honey is one of the most important quality indicators, as it affects viscosity, crystallization, and microbiological stability. In the study by Miłek et al. (2021) on the physicochemical properties of Polish honeys, the water content of nectar honeys ranged from 15.9 to 18.8%, while honeydew honeys exhibited slightly higher values, from 16.2 to 19.9% [46]. In comparison, the unadulterated honeys analyzed in our study showed similar levels (ca. 16–18%), whereas the addition of adulterants caused a marked increase, in many cases exceeding the regulatory threshold of 20%. Comparable ranges were reported by Majewska et al., (2019) [47] for Polish honeys, with moisture values between 15.8% and 20.4%. The authors noted that honeydew honeys generally contained less water than nectar honeys, a feature associated with their higher density and mineral content. For example, Baloš et al., (2019) examined 133 honey samples produced in Serbia in 2017 and 2018 and found water contents ranging from 14.0 to 20.8%, depending on the botanical origin [48]. Similarly to our observations, the lowest values were characteristic of honeydew honeys (15.2–16.2%), whereas linden honeys showed the highest moisture levels, occasionally exceeding the legal limit of 20%. An additional advantage of their study is the evaluation of year-to-year stability, which revealed no significant differences in average water content between production years, despite different meteorological conditions. This confirms that the water content in honey is relatively stable and primarily dependent on botanical origin rather than annual climatic variation.

### 3.4. Determination of Sugar Content

**Figure 4:**
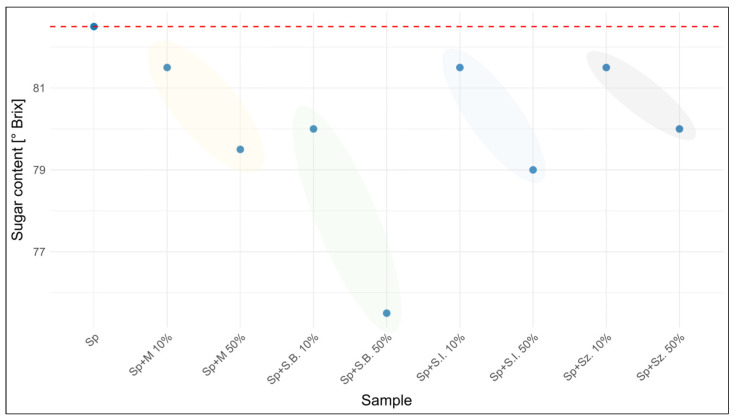
Percentage of sugar content in adulterated honeydew honey depending on the adulterant used and the level of adulteration.

Using a handheld refractometer designed for beekeepers, the sugar content in % Brix was determined in the tested samples. The obtained sugar content data are presented in Table 4 and in Figure 4 (honeydew honey). The sugar content of the analyzed honeys ranged from 81.5 to 82.5 °Brix, with the highest values observed for honeydew and forest honeys (82.5 °Brix) and the lowest for nectar–honeydew honey (81.5 °Brix). Among the adulterants, artificial honey exhibited a sugar content comparable to natural honeys (80.0 °Brix), whereas beet syrup showed a markedly lower value (68.5 °Brix). The addition of adulterants generally led to a decrease in sugar content, which was particularly pronounced in 50% mixtures. Beet syrup, due to its intrinsically low °Brix value, caused the strongest reductions across all honey types. By contrast, mixtures with artificial honey produced only minor changes, often within the natural variability of unadulterated honeys. This was especially evident for rapeseed honey, where even 50% adulteration with artificial honey yielded values (80.5 °Brix) close to the original sample (82.0 °Brix). These findings highlight that while the sugar content is a useful indicator for detecting adulteration with syrups of low °Brix, it is insufficient in cases where adulterants such as artificial honey closely mimic the sugar levels of authentic honeys, making adulteration more difficult to detect based on this parameter alone.

In the study by Terrab et al. (2004) on Moroccan honeys, the refractometric sugar content ranged from 79.0 to 83.2 °Brix, depending on the botanical origin, which is consistent with the values observed in our samples (81.5–82.5 °Brix) [49]. Similarly, Kamal & Klein (2011) [50] reported that Pakistani honeys typically exhibited sugar contents between 79.5 and 82.7 °Brix, again confirming that natural honeys fall within a relatively narrow range. By contrast, adulteration with syrups can significantly alter this parameter. Elflein & Raezke (2008) showed that the addition of beet and corn syrups caused a pronounced decrease in °Brix, in line with our findings, where beet syrup adulteration resulted in the strongest reductions [51]. In turn, Irudayaraj & Sivakesava (2001) noted that artificial honeys and inverted sugar syrups, due to their sugar levels closely matching those of authentic honey, are far more difficult to detect by simple refractometric analysis [52]. This is in agreement with our observations for the mixtures with artificial honey, where even 50% adulteration produced values indistinguishable from unadulterated honeys. Taken together, these results confirm that while sugar content analysis is a useful preliminary tool for identifying adulteration with low-°Brix syrups, it remains insufficient for detecting more sophisticated substitutes, highlighting the need for complementary analytical approaches.

The inverse relationship between water and sugar content in honey is well established, as these two parameters are closely interdependent. Higher concentrations of sugars, particularly glucose and fructose, result in lower water content due to the hygroscopic nature of honey and its high osmotic pressure. This balance determines the viscosity, crystallization tendency, and stability of honey. Similar relationships were also reported by Ratiu et al. (2020) [53] and Majewska et al. (2019) [47].

### 3.5. Determination of Electrical Conductivity

Electrical conductivity (EC) is a parameter widely applied in the assessment of honey quality and authenticity, being closely related to ash content as well as the concentration of mineral ions and organic acids. In the present study, EC values of the analyzed natural honeys ranged from 0.187 mS·cm^−1^ for rapeseed honey to 1.535 mS·cm^−1^ for forest honey (Table 4). Among the tested sugar-based adulterants, the lowest conductivity was observed for artificial honey (0.29 mS·cm^−1^), whereas invert syrup exhibited the highest value (31.6 mS·cm^−1^) (Table 4).

The addition of molasses and beet syrup resulted in a marked increase in EC, particularly in 1:1 mixtures, where the values exceeded several times those typical of natural honeys. In contrast, mixtures with invert syrup showed unexpectedly lower conductivity than would be predicted from the syrup alone. The addition of artificial honey generally reduced EC, with the exception of certain creamed honeys, for which a negligible increase was observed.

**Figure 5:**
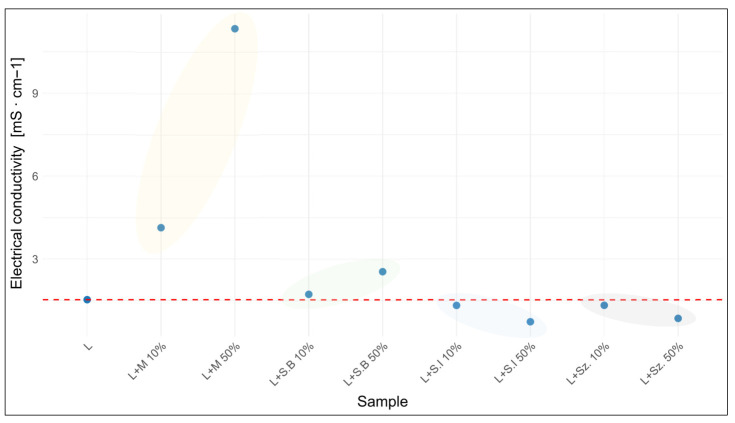
Value of electrical conductivity in adulterated forest honey depending on the adulterant used and the level of adulteration.

The extent of conductivity changes depended both on the adulterant and on the honey type. Forest honey was the most affected (Figure 5), while honeydew honey exhibited the smallest changes.

According to the Announcement of the Polish Ministry of Agriculture and Rural Development, an electrical conductivity value above 0.8 mS·cm^−1^ is characteristic of honeydew and nectar-honeydew honeys, whereas nectar honeys are required to exhibit values not exceeding this threshold [32]. Several examined samples and mixtures failed to meet these requirements. This was the case for honeydew and nectar–honeydew honeys adulterated with invert syrup (1:1 ratio), as well as the NS+Sz. 50% sample, which fell below the threshold. Importantly, forest honey itself did not comply with the limit for nectar honeys, most likely due to its high honeydew content. Among the forest honey mixtures, only the sample with 50% invert syrup remained within the acceptable range.

In the present study, the electrical conductivity of natural honeys varied according to their botanical origin, with the highest value recorded for forest honey and the lowest for rapeseed honey. This is consistent with the previous findings indicating that EC is strongly dependent on mineral composition and ash content [47,49,54,55,56].

Terrab et al., (2004) [49] characterized Spanish thyme honeys and reported a clear correlation between electrical conductivity, ash content, and mineral composition, with potassium being the predominant element. Their results confirmed that darker honeys, richer in minerals, exhibit higher EC values, whereas lighter nectar honeys show significantly lower values. Similarly, da Silva et al. (2016) [54] emphasized that EC is a reliable indicator of honey authenticity and quality, as it reflects not only the content of minerals but also contributions from organic acids and proteins. They also highlighted the strong positive correlation between ash, color intensity, and electrical conductivity, supporting its use as a classification and authentication tool. Comparable relationships were also observed by Kaczmarek et al. (2019), who reported a strong association between ash content, color intensity, and phenolic compounds in Polish honeys [55]. Since electrical conductivity is directly dependent on the mineral fraction, these findings further confirm that darker honeys—typically richer in ash and phenolics—exhibit higher EC values. Furthermore, the negative correlation between electrical conductivity and moisture content observed in this study is consistent with findings by Majewska et al. (2019), who reported that Polish honeydew and darker nectar honeys with lower water content exhibited significantly higher EC values [47]. This relationship reflects the dilution effect of water on the concentration of ionic species responsible for conductivity. Likewise, Wilczyńska et al. (2024) found strong correlations between the electrical properties of honey and its basic quality parameters, including moisture content and color, indicating that conductivity-related measurements can serve as fast and reliable indicators of mineralization and overall composition [56].

Thus, variations in EC, which are largely governed by the mineral fraction of honey, not only provide an effective criterion for distinguishing between nectar and honeydew honeys, but also represent a valuable tool for detecting adulteration; however, the interpretation of results should carefully account for possible matrix effects that may alter ionic mobility and conductivity values.

The effect of invert syrup addition on electrical conductivity proved to be particularly interesting. Although the syrup itself exhibited high conductivity values, its mixtures with honey showed markedly lower readings than would be expected from a simple proportional relationship. Since electrical conductivity reflects the concentration of ionizable mineral and organic constituents in diluted honey solutions, the addition of glucose and fructose-based syrups—lacking ionic species—results in an effective dilution of these components; as a consequence, the overall ionic strength of the system decreases, which directly affects the measured electrical conductivity, as electrical conductivity in honey is primarily determined by ion concentration in complex sugar-rich matrices [57,58]. In line with these considerations, the observed behaviour can be rationalized by two complementary mechanisms. First, a dilution effect occurs: invert syrup is composed almost exclusively of glucose and fructose and contributes negligible amounts of minerals or organic acids, which are the main carriers of electrical conductivity in honey [54]. Consequently, the relative share of mineral ions such as K^+^, Ca^2+^, and Mg^2+^, as well as phosphate and sulfate anions, decreases in the mixture, leading to lower conductivity. This interpretation is further supported by the elemental analysis, which clearly demonstrated that invert syrup contained only trace amounts of the ions responsible for electrical conductivity (Table 3). Second, a possible matrix effect should be considered. Organic acids, proteins, and phenolic compounds naturally present in honey may interact with cations and reduce their ionic mobility. In the presence of an excess of simple sugars from invert syrup, ionic balance and salt dissociation can be further altered, which may partially “mask” the contribution of minerals to electrical conductivity [49]. These combined effects may contribute to the unexpectedly low EC values observed in the mixtures. Overall, such behavior is consistent with previous observations indicating that electrical conductivity in honey is influenced not only by mineral content but also by the overall physicochemical matrix, which may lead to ambiguous EC-based interpretation in transitional systems [59].

### 3.6. Statistics and Chemometrics Analysis

**Figure 6:**
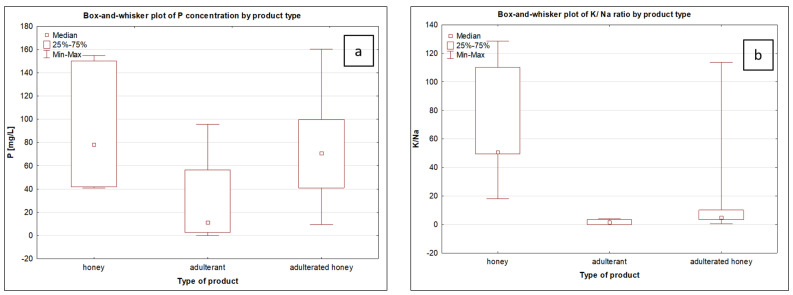

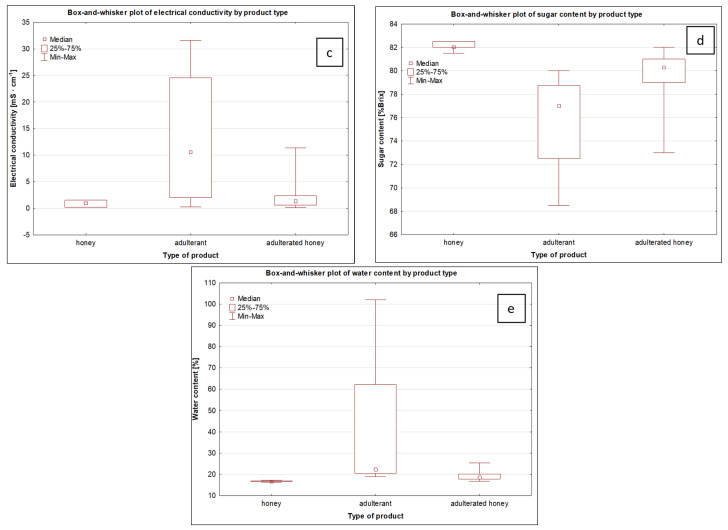
Box-and-whisker plots for P concentration (**a**), K/Na ratio (**b**), electrical conductivity (**c**), sugar content (**d**) and water content (**e**).

To enable a general comparison between natural honeys, sugar-based adulterants, and adulterated honeys, a univariate statistical analysis was performed using box-and-whisker plots for selected elemental and physicochemical parameters (Figure 6a–e). The analysis aimed to evaluate overall distribution patterns and central tendencies among the three product groups rather than to test statistical significance. Based on the median values, a consistent trend was observed for most parameters (P concentration, K/Na ratio and sugar content), with natural honeys showing the highest values, adulterants the lowest, and adulterated honeys occupying intermediate positions, reflecting the dilution effect caused by the addition of sugar products. This systematic shift in median values confirms the gradual loss of the characteristic honey composition with increasing adulterant contribution.

In contrast, for electrical conductivity and water content, a completely opposite order was observed, with the highest median values recorded for adulterants, the lowest for natural honeys, and adulterated honeys again exhibiting intermediate levels. The large dispersion observed for these two parameters in adulterants reflects the technological heterogeneity of sugar products. This opposite behaviour of sugar content in relation to electrical conductivity and water content is consistent with the fundamental physicochemical balance of honey and confirms that syrup addition disrupts its natural concentration equilibrium. Overall, the box-and-whisker analysis demonstrates clear distributional differences between natural honeys, adulterants and adulterated honeys, with statistically significant differences observed only for selected parameters, namely Na, K/Na ratio, water content and sugar content.

The Kruskal–Wallis test indicated that for most individual elements, differences between pure honey and adulterated samples were not statistically significant (*p* > 0.05). In contrast, statistically significant differences were observed for physicochemical parameters, including sugar content and water content, as well as for the K/Na ratio. Post hoc analysis confirmed significant differences between pure honey and sugar syrup, as well as between pure honey and honey adulterated with sugar syrup, for these variables (*p* < 0.05) (Table S5 in Supplementary Materials). These findings emphasize that physicochemical parameters and elemental ratios are more sensitive indicators of honey adulteration than absolute concentrations of individual elements.

**Figure 7:**
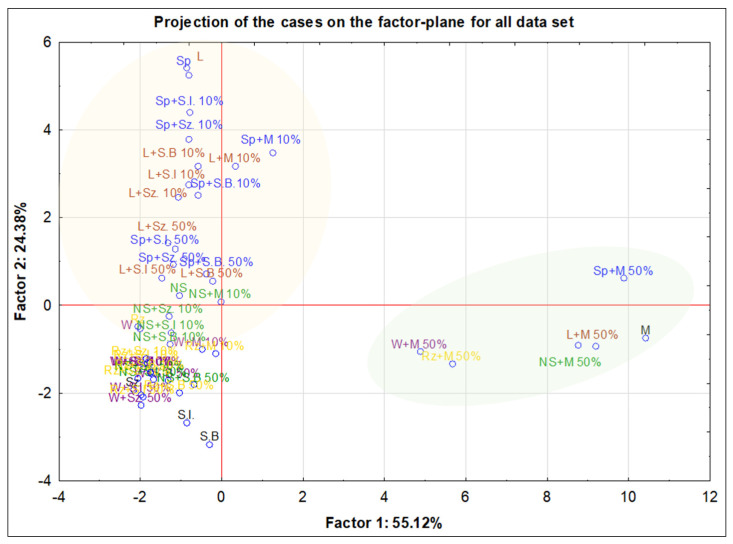
Projection of honey samples (with division into honey types), adulterant, and adulterated honey on the factor-plane for the entire data set.

To assess the multivariate structure of the dataset and the similarities between the analyzed samples, PCA was employed. Principal component analysis (PCA) demonstrated in Figure 7 revealed a clear separation between natural honeys, sugar-based adulterants and adulterated honeys in the space of the first two principal components, which together explained 79.5% of the total variance. A very strong influence of molasses (M) at the 50% addition level is clearly visible, manifested by a pronounced displacement of samples containing this additive away from the cluster of natural honeys and other adulterated variants. This indicates a substantial modification of both the elemental composition and the physicochemical properties of honey under the influence of a high proportion of molasses. Forest honey (L) and honeydew honey (Sp) exhibit a high degree of similarity in their elemental composition. All mixtures prepared with a 10% addition of sugar-based products to forest and honeydew honeys, as well as the corresponding unmodified honeys, formed a common cluster. The analogous mixtures at the 50% addition level were positioned below this group. This distribution indicates a substantial contribution of honeydew to the composition of forest honey. Nectar–honeydew honey (NS) was located between the clusters of honeydew and forest honeys and those of purely nectar honeys, namely rapeseed (Rz) and multi-floral (W) honeys. Rapeseed and multi-floral honeys showed similar elemental profiles and were characterized by generally low concentrations of most of the determined elements.

From a chemometric perspective, the PCA score plot reveals a continuous gradient of sample modification rather than strictly separated classes, reflecting the progressive transformation of the honey matrix with increasing adulterant contribution. The position of adulterated samples along the principal components follows a dose-dependent trend, particularly evident for the 50% variants, which are clearly shifted toward the region occupied by pure sugar-based additives. This confirms the high sensitivity of the multivariate model to compositional changes induced by adulteration. The relatively compact clustering of natural honeys indicates good internal consistency of their elemental and physicochemical profiles, whereas the wide dispersion of sugar-based adulterants reflects their pronounced compositional heterogeneity. Overall, the PCA model provides a clear multivariate visualization of similarities and differences among natural honeys, adulterated samples and pure sugar-based products, confirming the suitability of the applied chemometric approach for authenticity assessment.

Multivariate chemometric approaches have become a common and effective tool for the classification and authentication of honeys based on their mineral and physicochemical profiles. Multielemental data combined with PCA have been successfully applied to differentiate honeys according to their botanical origin, demonstrating the usefulness of chemometric tools for honey authentication [15,18,45]. Similarly, the application of ICP-based mineral profiling together with chemometric methods has enabled effective grouping and classification of honeys according to both botanical and geographical origin [44,60,61,62]. Multielemental profiling combined with chemometrics has been shown to effectively detect compositional deviations associated with honey adulteration [1,6,63]. Altogether, these findings support the validity of applying PCA-based multivariate analysis in the present work for the characterization of natural honeys, sugar-based adulterants and adulterated honeys.

To further verify the relationships observed in the PCA results, hierarchical cluster analysis (HCA) of the elemental variables was additionally performed using Ward’s method and Euclidean distances (Figure S1, Supplementary Materials). The dendrogram confirmed the existence of two main groups of strongly associated elements, corresponding to the macroelement fraction and the group of trace elements. This result is consistent with both the correlation analysis and the PCA outcomes, further supporting the robustness of the multivariate structure of the dataset.

## 4. Conclusions

Adulteration with sugar syrups markedly altered both the elemental profile and physicochemical properties of honey. The K/Na ratio emerged as a highly informative authenticity marker, effectively differentiating pure from adulterated samples under the applied experimental conditions. Beet molasses and beet syrup caused the strongest disturbances in mineral balance and conductivity, while invert syrup produced smaller changes. Artificial honey closely imitated natural moisture and sugar levels, masking adulteration in basic tests. °Brix measurements effectively revealed low-sugar syrups, and electrical conductivity provided complementary evidence linked to mineral composition. In contrast, electrical conductivity and water content showed an inverse relationship, with higher values of one parameter corresponding to lower values of the other, reflecting the natural physicochemical balance of honey and its disruption by syrup addition. Statistical and chemometric analyses complemented the interpretation of elemental and physicochemical data. PCA revealed a continuous gradient of sample transformation from natural honeys toward pure sugar-based products, depending on both the type and level of additive. A particularly strong chemometric effect was observed for 50% molasses additions, which caused pronounced displacement of samples in the multivariate space. Overall, the integration of elemental profiling, physicochemical characterization and chemometric analysis provides a robust, complementary strategy for honey authenticity assessment, enhancing the detection of compositional deviations associated with adulteration even when individual parameters show limited diagnostic power.

## Figures and Tables

**Table 1:** Types of honey and food additives used in the research.

Type of Tested Product	Code	Origin/Manufacturer
Honeydew honey	Sp	A home apiary in the Świętokrzyskie Voivodeship
Honey from forest areas (nectar/nectar-honeydew)	L	A home apiary in the Świętokrzyskie Voivodeship
Nectar-honeydew honey	NS	‘Mazurskie miody’ Apiary, Tomaszkowo, Warmińsko-mazurskie Voivodeship, Poland
Creamed rapeseed honey (nectar)	Rz	‘Dar Natury’ Apiary, Szadek, Łódzkie Voivodeship, Poland
Creamed spring multiflower honey (nectar)	W	‘Dar Natury’ Apiary, Szadek, Łódzkie Voivodeship
Invert syrup	S.I.	‘Mazurskie miody’ Apiary, Tomaszkowo, Warmińsko-mazurskie Voivodeship
Thick beetroot syrup	S.B.	Product obtained directly from Krajowa Grupa Spożywcza S.A., “Sugar Factory Dobrzelin” Branch in Dobrzelin, Poland (acquired during the 2024 sugar campaign)
Beet molasses	M	Product obtained directly from Krajowa Grupa Spożywcza S.A., “Sugar Factory Dobrzelin” Branch in Dobrzelin (acquired during the 2024 sugar campaign)
Artificial liquid honey	Sz	‘Rydełkiewicz S.C.’, Gola Górowska, Dolnośląskie Voivodeship, Poland

**Table 2:** ICP-OES (Thermo Scientific, ICAP 7000 series, MA, USA) parameters and measurement conditions.

Parameter and Accessories	ICP-OES
Number of replicates	3
Carrier gas	Argon
Plasma gas flow rate [L·min^−1^]	11.5
Auxiliary gas flow rate [L·min^−1^]	0.5
Nebulization gas flow rate [L·min^−1^]	0.5
Nebulizer gas pressure [kPa]	280
Torch	Quartz
Nebulizer	Concentric quartz
Generator power [W]	1150
Internal standard	Yb

**Table 3:** Ratios of K/Na in pure honeys and their adulterated variants.

Honey	Pure	S.I. 10%	S.I. 50%	Sz 10%	Sz 50%	S.B. 10%	S.B. 50%	M 10%	M 50%
Sp	128.6	56.08	51.12	12.06	11.57	6.29	6.290	6.130	3.280
L	110.1	70.64	42.81	28.90	12.21	12.33	6.270	5.650	3.260
W	50.56	3.630	3.630	4.690	0.490	4.800	4.130	3.500	3.270
NS	18.16	4.760	3.860	2.260	7.380	4.100	4.100	4.830	3.230
Rz	49.59	3.060	3.530	1.080	4.400	4.260	4.260	1.500	3.330

**Table 4:** Results of the analysis of water content in tested samples and their mixtures.

Sample	Water Content [%]	Sugar Content [% Brix]	Electrical Conductivity [mS·cm^−1^]
Sp	16.7	82.5	1.55
Sp+S.B. 10%	19.4	80.0	1.72
Sp+S.B. 50%	23.2	75.5	2.49
Sp+S.I. 10%	17.8	81.5	1.35
Sp+S.I. 50%	20.6	79.0	0.78
Sp+Sz. 10%	17.4	81.5	1.38
Sp+Sz. 50%	18.9	80.0	0.85
Sp+M 10%	17.4	81.5	3.08
Sp+M 50%	18.2	79.5	8.78
NS	17.2	81.5	0.94
NS+S.B 10%	20.2	79.0	1.23
NS+S.B 50%	25.4	73.0	2.28
NS+S.I 10%	18.6	80.5	0.81
NS+S.I 50%	20.2	79.0	0.54
NS+Sz. 10%	17.8	81.0	0.82
NS+Sz. 50%	18.2	80.5	0.63
NS+M 10%	18.6	81.0	2.65
NS+M 50%	22.6	77.0	10.21
L	16.2	82.5	1.54
L+S.B 10%	18.3	80.5	1.73
L+S.B 50%	23.5	75.0	2.55
L+S.I 10%	17.0	81.0	1.33
L+S.I 50%	20.5	78.5	0.74
L+Sz. 10%	16.7	82.0	1.34
L+Sz. 50%	18.5	80.5	0.86
L+M 10%	18.6	81.0	4.15
L+M 50%	23.4	77.0	11.35
W	16.6	82.0	0.20
W+S.B 10%	17.8	80.5	0.49
W+S.B 50%	22.6	76.5	1.65
W+S.I 10%	17.0	80.5	0.21
W+S.I 50%	19.0	78.0	0.15
W+Sz. 10%	17.4	81.0	0.22
W+Sz. 50%	18.6	79.5	0.25
W+M 10%	17.0	81.5	2.03
W+M 50%	19.8	79.0	8.50
Rz	17.0	82.0	0.19
Rz+S.B 10%	17.8	80.0	0.64
Rz+S.B 50%	22.2	76.5	1.78
Rz+S.I 10%	17.8	81.0	0.18
Rz+S.I 50%	19.0	79.0	0.14
Rz+Sz. 10%	17.1	80.5	0.20
Rz+Sz. 50%	17.8	80.5	0.23
Rz+M 10%	17.4	81.0	2.20
Rz+M 50%	18.6	79.5	9.05
S.B	<27.0	68.5	3.75
S.I.	22.6	76.5	31.6
Sz.	19.0	80.0	0.29
M	21.8	77.5	17.47

## Data Availability

The original contributions presented in the study are included in the article/Supplementary Material, further inquiries can be directed to the corresponding author.

## Supplementary Materials

The following supporting information can be downloaded at: https://www.mdpi.com/article/10.3390/foods15030562/s1, Figure S1: Hierarchical cluster dendrogram of the analyzed elements obtained using Ward’s method and Euclidean distance, illustrating the similarity structure among elemental variables in all honey samples; Table S1: Overview of selected studies employing ICP-based techniques for honey analysis; Table S2: Concentrations of individual elements in the analyzed samples, expressed in mg/L; Table S3: Basic validation parameters obtained for each analyte for the developed method (n, number of standards in three replicates, R2, coefficient of determination, CRM’s values); Table S4: Pearson correlation matrix for all analyzed elements in honey and adulterated samples; Table S5: Results of post hoc Dunn’s test for selected physicochemical parameters and elemental ratios.
