# Supplementary material for: Analysis of Changes in Selected Physicochemical Parameters and Elemental Composition of Honey as a Result of Adulteration with Sugar Additives
Source: Foods. 2026 Feb 5;15(3):562. doi: 10.3390/foods15030562 (PMC12897118; doi:10.3390/foods15030562)
Supplement: Supplementary file 1 [file foods-15-00562-s001.zip › foods-4078477-supplementary.pdf]

Table S1. Overview of selected studies employing ICP-based techniques for honey analysis

Aim of the study	Main application	Studied samples	Sample preparation	Analytical technique	Analyzed elements	Ref
Determination of elemental composition of bee products from diverse botanical sources and assessment of potential health risks associated with their consumption.	Botanical origin characterization and consumer safety	143 bee product samples: 111 honeys (nectar, honeydew, nectar–honeydew), 14 pollen, 9 propolis, and 9 bee bread samples, mainly from Poland (10 voivodeships), with a few samples from other European countries	0.5 g of sample mineralized with 5 mL of 65% HNO_3 using microwave-assisted digestion; digests diluted to 50 mL with deionized water; ytterbium (Yb) used as an internal standard	ICP-OES	Ag, Al, Ba, Ca, Cd, Co, Cr, Cu, Fe, K, Mg, Mn, Mo, Na, Ni, P, Pb, S, Sb, Sn, Sr, Ti, V, Zn	[15]
Identification of elemental signatures for New Zealand and international honeys and categorization of New Zealand honey based on elemental profiles, regardless of botanical origin	Geographical origin authentication	352 monofloral and multifloral nectar honeys from 34 locations	200 mg of honey was heated and centrifuged, sample taken and added 0.2 ml nitric acid, 0.1 ml hydrogen peroxide, and filled to 6 ml with Type 1 water	ICP-MS	K, Na, Ca, Mg, Al, B, Mn, Rb, Zn, Fe, Cu, Sr, Ba, Co, Cr, Cs, Ni, Pb, Tl	[16]
Development of a simple analytical method for quantifying 39 elements in	Analytical method development	5 honey samples from various	200 mg of honey was heated and centrifuged, sample taken and added	ICP-MS	K, Na, Ca, Mg, Fe, Zn, Cu, Mn, Se, Pb, Cd, As, Hg, Al, Cr, Ni	[17]

small mass samples of bees, honey, and pollen using ICP-MS		sources in New Zealand	0.2 ml nitric acid, 0.1 ml hydrogen peroxide, and filled to 6 ml with Type 1 water			
Determination of multi-element content and physicochemical properties of Malaysian honeys and analysis of elemental markers of botanical and geographical origin	Geographical and botanical origin characterization	6 honeys from Malaysia: tualang, gelam, acacia, and 3 forest honeys	1 g of honey mineralized with 10 ml 65% HNO3 by heating, then diluted to 50 ml with double-deionized water	ICP-AES, ICP-MS	K, Na, Mg, Fe, Zn, Mn, Al, Cr, Rb, Cu, Sr, In, Ni, Se, Cs, Ba, Ag, U	[18]
Correlation of honey mineral composition with botanical and geographical origin, and risk assessment of toxic elements for consumer protection	Geographical origin authentication	39 nectar honeys: orange, lemon, chestnut, eucalyptus, acacia, sulla, wildflower from Sicily and Calabria	0.4 g sample with 7 ml 65% HNO3, 1 ml 30% H2O2, 1 ml internal Re standard, diluted to 25 ml with ultrapure water	ICP-OES, ICP-MS	K, Ca, Mg, Na, Al, Zn, Fe, Mn, Cu, Cr, Ni, Se, Pb, Cd, As	[19]
Characterization of multi-element profiles of monofloral and polyfloral honey and their differentiation based on floral origin	Botanical origin characterization	83 honeys from Latvia: multifloral, linden, blueberry, raspberry-blackberry, mint, raspberry, heather, rapeseed	1 g honey diluted with 4 ml ultrapure water, 5 ml 69% HNO3, 1 ml 30% H2O2, 2-step microwave digestion, added Pt as internal standard	ICP-MS	K, Na, Ca, Mg, Al, Ba, Cd, Co, Cr, Cu, Fe, Li, Mn, Mo, Ni, Pb, Rb, Sr, Ti, Tl, V, Zn, As, Sb, Sn, U, Se, Bi, Cs, Zr	[20]

Development and validation of an ICP-MS method for trace and toxic element analysis in monofloral honeys	Botanical origin authentication and safety assessment	133 honeys: 33 asphodel, 30 eucalyptus, 31 strawberry tree, 39 thistle	0.5 g honey with 5 ml 65% HNO3 and 2 ml 30% H2O2, diluted with deionized water to 25 ml	ICP-MS	K, Na, Ca, Mg, Fe, Zn, Mn, Cu, Co, Ni, Cr, Mo, Pb, Cd, As, Hg, Al, Sb, Tl, Sn, V	[21]
Characterization of Italian multifloral honeys from Lazio based on mineral profile and quality parameters for assessing environmental influence and geographical origin	Geographical origin discrimination and environmental assessment	40 multifloral honeys from 5 provinces in Lazio, Italy	0.8 g honey with 3.5 ml 70% HNO3 and 30% H2O2, microwave digestion system (Milestone, Start D), final solution diluted to 15 ml with deionized water	ICP-OES	K, Na, Ca, Mg, P, S, Al, As, Cd, Pb, Fe, Zn, Cu, Mn, Co, Ni, Cr, V, U, Tl, Sn, Sb, Be, Li, Sr, Ba, B, Si, Se	[22]

[15] Gajek, M.; Wysocki, P.; Mordaka, K.; Jóźwik, M. I. S. *Elemental composition of bee products from diverse botanical sources: Implications for consumer safety*. Journal of Food Composition and Analysis, **2025**, *147*.

[16] Grainger, M.N.C.; Klaus, H.; Hewi, N.; Gan, H.; French, A.D. *Graphical Discrimination of New Zealand Honey from International Honey Using Elemental Analysis.* Biol. Trace Elem. Res. **2024**, *202*(2), 754–764. https://doi.org/10.1007/s12011-023-03960-2

[17] Grainger, M.N.C.; Hewi, N.; Gan, H.; French, A.D. *Optimised Approach for Small Mass Sample Preparation and Elemental Analysis of Bees and Bee Products by Inductively Coupled Plasma Mass Spectrometry.* Talanta **2020**, *214*, 120858. https://doi.org/10.1016/j.talanta.2020.120858

[18] Abdul-Rahaman, N.; Aziz, R.; Chua, L.S.; Sarmidi, M.R. *Multi-Elemental Composition and Physical Properties of Honey Samples from Malaysia.* Food Chem. **2012**, *135*(3), 880–887. https://doi.org/10.1016/j.foodchem.2012.05.110

[19] Di Bella, G.; Lo Turco, V.; Potortì, A.G.; Bua, G.D.; Fede, M.R.; Dugo, G. Geographical discrimination of Italian honey by multi-elemental analysis with a chemometric approach. *J. Food Compos. Anal.* **2015**, *44*, 25–35. https://doi.org/10.1016/j.jfca.2015.03.006

[20] Labsvards, K.D.; Borisova, A.; Kokina, K.; Bertins, M.; Naumenko, J.; Rudovica, V.; Viksna, A. *Multi-Element Profile Characterization of Monofloral and Polyfloral Honey from Latvia*. Foods **2023,** *12*(22), 4091. https://doi.org/10.3390/foods12224091

[21] Mara, A.; Deidda, S.; Caredda, M.; Ciulu, M.; Deroma, M.; Farinini, E.; Floris, I.; Langasco, I.; Leardi, R.; Pilo, M.I.; *et al.* Multi-Elemental Analysis as a Tool to Ascertain the Safety and the Origin of Beehive Products: Development, Validation, and Application of an ICP-MS Method on Four Unifloral Honeys Produced in Sardinia, Italy. *Molecules* **2022**, *27*, 2009. https://doi.org/10.3390/molecules27062009

[22] Conti, M.E.; Botrè, F.; Ciarrocca, M.; Fanali, S.; Giardina, C.; Poma, G.; Menci, C.; Paduano, E. *Characterization of Italian Multifloral Honeys on the Basis of Their Elemental Composition and Some Quality Parameters.* J. Food Compos. Anal. **2018**, *74*, 1–17. https://doi.org/10.1016/j.jfca.2018.07.007

Table S2. Concentrations of individual elements in the analyzed samples, expressed in mg/kg

Sample	Al 396.152	Ca 393.366	Co 228.616	Cu 324.754	Fe 238.204	K 766.490	Mg 279.553	Mn 257.610	Na 589.592	Ni 231.604	P 177.495	S 180.731	Sr 407.771	Ti 334.941	Zn 213.856
Sp	35.46 ± 0.007	35.83 ± 0.002	<LOD	1.201 ± 0.000	<LOD	3604 ± 0.147	87.82 ± 0.005	5.905 ± 0.000	28.02 ± 0.006	0.767 ± 0.001	150.2 ± 0.006	112.1 ± 0.003	<LOD	<LOD	2.802 ± 0.007
NS	15.05 ± 0.001	60.78 ± 0.006	<LOD	0.613 ± 0.001	<LOD	1291 ± 0.057	26.41 ± 0.001	2.860 ± 0.000	271.1 ± 0.017	<LOD	78.32 ± 0.004	48.37 ± 0.000	<LOD	<LOD	9.909 ± 0.001
L	38.20 ± 0.004	39.60 ± 0.004	<LOD	1.320 ± 0.000	<LOD	3665 ± 0.315	89.80 ± 0.007	6.091 ± 0.001	33.30 ± 0.008	0.778 ± 0.000	155.0 ± 0,002	118.9 ± 0.001	<LOD	<LOD	<LOD
Rz	20.58 ± 0.001	53.26 ± 0.000	<LOD	<LOD	<LOD	206.2 ± 0.027	13.81 ± 0.000	<LOD	44.49 ± 0.019	<LOD	40.86 ± 0.001	27.18 ± 0.004	<LOD	<LOD	<LOD
W	19.32 ± 0.001	52.93 ± 0.001	<LOD	<LOD	<LOD	213.6 ± 0.059	14.02 ± 0.000	<LOD	43.78 ± 0.011	<LOD	41.78 ± 0.001	95.58 ± 0.005	<LOD	<LOD	<LOD
S.I.	18.46 ± 0.001	16.83 ± 0.001	<LOD	<LOD	<LOD	4.657 ± 0.023	0.551 ± 0.000	<LOD	34.76 ± 0.001	<LOD	5.475 ± 0.001	<LOD	<LOD	<LOD	3.606 ± 0.001
Sz	19.41 ± 0.004	9.099 ± 0.001	<LOD	<LOD	<LOD	37.03 ± 0.034	<LOD	<LOD	242.5 ± 0.023	<LOD	<LOD	11.50 ± 0.001	<LOD	<LOD	<LOD
S.B.	13.71 ± 0.001	71.52 ± 0.001	<LOD	<LOD	<LOD	5411 ± 0.289	4.996 ± 0.000	6.206 ± 0.001	1359 ± 0.114	<LOD	17.32 ± 0.000	290.7 ± 0.002	0.736 ± 0.000	<LOD	3.786 ± 0.000
M	9.423 ± 0.001	895.5 ± 0.141	0.801 ± 0.000	0.916 ± 0.000	21.69 ± 0.001	30596 ± 0.361	38.63 ± 0.001	6.180 ± 0.001	9461 ± 0.199	3.624 ± 0.000	95.64 ± 0.005	2026 ± 0.002	6.295 ± 0.000	0.458 ± 0.000	20.94 ± 0.000
W+ S.I. 10%	12.99 ± 0.001	50.52 ± 0.004	<LOD	<LOD	<LOD	234.3 ± 0.006	13.61 ± 0.001	<LOD	26.72 ± 0.017	<LOD	41.85 ± 0.002	27.47 ± 0.001	<LOD	<LOD	<LOD
W+ S.I. 50%	13.25 ± 0.001	26.42 ± 0.001	<LOD	<LOD	<LOD	128.8 ± 0.011	6.505 ± 0.000	<LOD	35.50 ± 0.006	<LOD	21.25 ± 0.000	12.15 ± 0.001	<LOD	<LOD	<LOD
W+Sz 10%	13.21 ± 0.001	51.12 ± 0.008	<LOD	<LOD	<LOD	214.3 ± 0.045	13.43 ± 0.001	<LOD	45.73 ± 0.001	<LOD	41.20 ± 0.003	27.31 ± 0.001	<LOD	<LOD	<LOD
W+Sz 50%	9.736 ± 0.000	23.80 ± 0.002	<LOD	<LOD	<LOD	85.83 ± 0.023	4.647 ± 0.000	<LOD	173.6 ± 0.013	<LOD	9.361 ± 0.000	15.47 ± 0.000	<LOD	<LOD	<LOD
W+ S.B. 10%	11.98 ± 0.001	53.86 ± 0.004	<LOD	<LOD	<LOD	855.9 ± 0.063	13.11 ± 0.000	1.012 ± 0.000	178.4 ± 0.018	<LOD	42.39 ± 0.001	61.20 ± 0.001	<LOD	<LOD	<LOD
W+ S.B. 50%	13.86 ± 0.000	61.20 ± 0.005	<LOD	<LOD	<LOD	2676 ± 0.180	9.708 ± 0.001	3.151 ± 0.000	648.7 ± 0.025	<LOD	31.28 ± 0.001	151.1 ± 0.003	0.407 ± 0.000	<LOD	<LOD
W+M 10%	9.547 ± 0.000	157.1 ± 0.015	<LOD	0.534 ± 0.000	6.893 ± 0.000	3624 ± 0.095	19.40 ± 0.001	0.962 ± 0.000	1034 ± 0.009	<LOD	52.12 ± 0.003	249.5 ± 0.008	0.855 ± 0.000	<LOD	3.206 ± 0.000
W+M 50%	10.57 ± 0.001	523.6 ± 0.004	0.448 ± 0.000	0.448 ± 0.000	11.82 ± 0.001	17516 ± 0.468	27.62 ± 0.001	3.697 ± 0.000	5354 ± 0.121	2.091 ± 0.000	73.23 ± 0.001	1160 ± 0.008	3.697 ± 0.000	0.200 ± 0.001	11.65 ± 0.000
NS+ S.I. 10%	15.36 ± 0.002	47.38 ± 0.005	<LOD	0.299 ± 0.000	<LOD	975.1 ± 0.070	19.66 ± 0.001	2.090 ± 0.000	203.2 ± 0.219	<LOD	61.69 ± 0.000	35.19 ± 0.002	<LOD	<LOD	9.176 ± 0.000
NS+ S.I. 50%	11.71 ± 0.002	26.74 ± 0.000	<LOD	<LOD	<LOD	538.3 ± 0.024	11.03 ± 0.001	1.197 ± 0.000	113.1 ± 0.009	<LOD	36.29 ± 0.001	20.51 ± 0.001	<LOD	<LOD	3.694 ± 0.000
NS+ Sz 10%	16.60 ± 0.001	66.68 ± 0.004	<LOD	0.407 ± 0.001	<LOD	1001 ± 0.028	22.41 ± 0.001	2.456 ± 0.000	259.4 ± 0.016	<LOD	71.12 ± 0.004	42.55 ± 0.002	<LOD	<LOD	5.656 ± 0.000
NS+ Sz 50%	13.08 ± 0.001	26.29 ± 0.002	<LOD	<LOD	<LOD	534.8 ± 0.017	10.51 ± 0.001	1.195 ± 0.000	236.8 ± 0.013	<LOD	32.63 ± 0.001	24.05 ± 0.002	<LOD	<LOD	2.589 ± 0.000
NS+ S.B. 10%	15.11 ± 0.001	46.86 ± 0.005	<LOD	0.302	<LOD	1856 ± 0.018	17.35 ± 0.001	1.911 ± 0.000	251.4 ± 0.025	<LOD	54.37 ± 0.004	135.85 ± 0.000	<LOD	<LOD	6.034 ± 0.000
NS+ S.B. 50%	11.94 ± 0.001	66.34 ± 0.004	<LOD	<LOD	<LOD	3935 ± 0.163	12.47 ± 0.001	4.968 ± 0.001	960.0 ± 0.097	<LOD	41.26 ± 0.001	203.3 ± 0.001	0.517 ± 0.000	<LOD	4.968 ± 0.000

NS+M 10%	14.79 ± 0.000	175.8 ± 0.001	<LOD	0.555 ± 0.001	<LOD	5261 ± 0.236	28.35 ± 0.001	3.329 ± 0.000	1089 ± 0.047	0.629 ± 0.001	87.51 ± 0.012	308.4 ± 0.036	0.999 ± 0.000	<LOD	8.654 ± 0.001
NS+M 50%	7.565 ± 0.000	815.8 ± 0.047	0.674 ± 0.000	0.674 ± 0.000	18.93 ± 0.000	27081 ± 1.390	36.80 ± 0.001	5.842 ± 0.000	8387 ± 0.610	3.333 ± 0.000	96.59 ± 0.004	1793 ± 0.039	5.618 ± 0.000	0.337 ± 0.000	19.10 ± 0.001
L+S.I. 10%	23.45 ± 0.004	34.66 ± 0.001	<LOD	1.135 ± 0.000	<LOD	2447 ± 0.025	58.95 ± 0.001	4.229 ± 0.001	34.64 ± 0.004	0.690 ± 0.000	111.6 ± 0.005	77.51 ± 0.001	<LOD	<LOD	4.848 ± 0.000
L+S.I. 50%	21.06 ± 0.000	26.31 ± 0.001	<LOD	0.609 ± 0.000	<LOD	1494 ± 0.081	36.21 ± 0.001	2.539 ± 0.000	34.90 ± 0.014	0.280 ± 0.000	69.64 ± 0.004	45.45 ± 0.000	<LOD	<LOD	<LOD
L+Sz 10%	25.31 ± 0.001	27.49 ± 0.002	<LOD	1.007 ± 0.000	<LOD	2564 ± 0.025	62.29 ± 0.001	4.431 ± 0.000	88.73 ± 0.005	0.470 ± 0.001	115.2 ± 0.003	82.33 ± 0.001	<LOD	<LOD	<LOD
L+Sz 50%	23.38 ± 0.003	24.96 ± 0.002	<LOD	1.213 ± 0.000	<LOD	1837 ± 0.042	45.33 ± 0.002	3.234 ± 0.000	150.4 ± 0.001	<LOD	82.13 ± 0.001	62.61 ± 0.001	<LOD	<LOD	<LOD
L+ S.B. 10%	32.87 ± 0.001	42.50 ± 0.004	<LOD	1.108 ± 0.000	<LOD	3928 ± 0.173	70.85 ± 0.004	6.244 ± 0.000	318.5 ± 0.044	0.671 ± 0.000	136.9 ± 0.004	159.0 ± 0.004	<LOD	<LOD	1.719 ± 0.000
L+ S.B. 50%	21.13 ± 0.000	81.14 ± 0.001	<LOD	0.624 ± 0.000	<LOD	4518 ± 0.046	43.91 ± 0.000	6.132 ± 0.000	720.9 ± 0.010	0.589 ± 0.001	86.96 ± 0.001	411.3 ± 0.018	0.416 ± 0.000	<LOD	2.702 ± 0.000
L+M 10%	31.34 ± 0.000	168.3 ± 0.007	<LOD	1.273 ± 0.000	6.530 ± 0.000	7159 ± 0.519	82.72 ± 0.006	6.155 ± 0.001	1267 ± 0.089	1.238 ± 0.001	160.1 ± 0.004	361.1 ± 0.006	1.061 ± 0.000	<LOD	11.78 ± 0.000
L+M 50%	7.822 ± 0.000	871.0 ± 0.023	0.697 ± 0.000	0.697 ± 0.001	20.67 ± 0.000	28783 ± 2.625	41.30 ± 0.002	6.157 ± 0.000	8837 ± 1.073	2.189 ± 0.000	102.9 ± 0.001	1908 ± 0.026	5.925 ± 0.000	0.465 ± 0.000	18.85 ± 0.000
Sp+ S.I. 10%	28.60 ± 0.003	30.62 ± 0.001	<LOD	1.134 ± 0.000	<LOD	3212 ± 0.081	78.73 ± 0.001	5.568 ± 0.000	28.25 ± 0.001	0.687 ± 0.000	150.8 ± 0.006	102.1 ± 0.011	<LOD	<LOD	2.972 ± 0.001
Sp+ S.I. 50%	18.34 ± 0.000	18.21 ± 0.001	<LOD	0.614 ± 0.000	<LOD	1642 ± 0.022	39.43 ± 0.001	6.864 ± 0.000	29.28 ± 0.015	<LOD	78.41 ± 0.000	54.26 ± 0.001	<LOD	<LOD	3.051 ± 0.000
Sp+ Sz 10%	33.26 ± 0.000	34.85 ± 0.001	<LOD	1.114 ± 0.000	<LOD	3092 ± 0.151	75.22 ± 0.004	5.268 ± 0.000	60.48 ± 0.033	0.675 ± 0.001	144.7 ± 0.001	102.7 ± 0.002	<LOD	<LOD	2.167 ± 0.000
Sp+ Sz 50%	18.74 ± 0.001	18.61 ± 0.001	<LOD	0.700 ± 0.001	<LOD	1616 ± 0.091	38.67 ± 0.001	6.801 ± 0.000	134.0 ± 0.033	<LOD	74.10 ± 0.006	56.97 ± 0.001	<LOD	<LOD	0.555 ± 0.000
Sp+ S.B. 10%	26.31 ± 0.001	43.10 ± 0.000	<LOD	0.935 ± 0.000	<LOD	3968 ± 0.084	67.25 ± 0.001	6.127 ± 0.001	343.0 ± 0.015	0.692 ± 0.000	131.4 ± 0.000	158.3 ± 0.004	<LOD	<LOD	1.295 ± 0.000
Sp+ S.B. 50%	20.98 ± 0.003	52.52 ± 0.004	<LOD	0.722 ± 0.001	<LOD	4358 ± 0.168	43.90 ± 0.004	6.087 ± 0.000	692.5 ± 0.039	0.585 ± 0.001	89.52 ± 0.003	200.3 ± 0.001	0.413 ± 0.000	<LOD	2.992 ± 0.000
Sp+M 10%	28.43 ± 0.000	132.5 ± 0.011	<LOD	1.253 ± 0.001	<LOD	6654 ± 0.775	81.57 ± 0.008	6.158 ± 0.000	1086 ± 0.103	1.113 ± 0.000	133.3 ± 0.013	338.8 ± 0.023	0.835 ± 0.000	<LOD	4.175 ± 0.001
Sp+M 50%	13.99 ± 0.000	830.1 ± 0.086	0.692 ± 0.000	1.384 ± 0.000	20.08 ± 0.001	27527 ± 0.469	46.64 ± 0.001	6.342 ± 0.000	8397 ± 0.020	3.421 ± 0.000	113.7 ± 0.002	1848 ± 0.006	5.650 ± 0.000	0.461 ± 0.001	32.86 ± 0.001
Rz+ S.I. 10%	15.82 ± 0.001	45.17 ± 0.002	<LOD	<LOD	<LOD	217.4 ± 0.033	13.01 ± 0.001	<LOD	35.32 ± 0.001	<LOD	43.15 ± 0.001	20.17 ± 0.000	<LOD	<LOD	3.458 ± 0.000
Rz+ S.I. 50%	9.548 ± 0.000	37.60 ± 0.002	<LOD	<LOD	<LOD	119.9 ± 0.033	7.325 ± 0.000	<LOD	39.22 ± 0.005	<LOD	25.35 ± 0.003	15.07 ± 0.001	<LOD	<LOD	<LOD
Rz+ Sz 10%	19.37 ± 0.001	51.34 ± 0.004	<LOD	<LOD	<LOD	206.4 ± 0.008	12.56 ± 0.001	<LOD	58.46 ± 0.013	<LOD	40.67 ± 0.003	28.11 ± 0.003	<LOD	<LOD	3.355 ± 0.000
Rz+ Sz 50%	16.61 ± 0.000	29.14 ± 0.001	<LOD	<LOD	<LOD	126.4 ± 0.041	6.902 ± 0.000	<LOD	117.4 ± 0.004	<LOD	22.57 ± 0.002	18.46 ± 0.001	<LOD	<LOD	<LOD
Rz+ S.B. 10%	11.92 ± 0.001	50.60 ± 0.001	<LOD	<LOD	<LOD	978.1 ± 0.016	11.94 ± 0.001	1.219 ± 0.000	222.1 ± 0.021	<LOD	40.71 ± 0.000	66.19 ± 0.005	<LOD	<LOD	<LOD
Rz+ S.B. 50%	8.489 ± 0.001	82.61 ± 0.007	<LOD	<LOD	<LOD	2843 ± 0.143	7.003 ± 0.001	3.361 ± 0.000	667.5 ± 0.025	<LOD	33.68 ± 0.002	165.0 ± 0.005	0.509 ± 0.000	<LOD	3.334 ± 0.000
Rz+M 10%	20.11 ±	372.1 ±	<LOD	<LOD	6.856 ±	3516 ±	19.12 ±	1.003 ±	2343 ±	<LOD	51.13 ±	225.2 ±	1.784 ±	<LOD	3.121 ±

	0.021	0.031			0.000	0.021	0.001	0.000	0.084		0.001	0.000	0.000		0.000
Rz+M 50%	4.197 ± 0.000	544.0 ± 0.050	0.462 ± 0.000	0.462 ± 0.000	12.77 ± 0.000	18184 ± 0.497	27.78 ± 0.000	3.812 ± 0.001	5463 ± 0.020	2.156 ± 0.000	81.06 ± 0.004	1196 ± 0.018	3.697 ± 0.000	0.399 ± 0.000	13.29 ± 0.001
Min	4.197	9.099	0.448	0.299	6.530	4.657	0.551	0.962	26.72	0.28	5.475	11.5	0.407	0.2	0.555
Max	38.20	895.5	0.801	1.384	21.69	30596	89.80	6.864	9461	3.624	160.1	2026	6.295	0.465	32.86
Mean	17.80	144.9	0.629	0.831	14.03	4950	32.53	4.237	1259	1.334	72.88	306.3	2.319	0.387	7.039
Median	15.82	50.60	0.683	0.711	12.77	2447	24.41	4.431	222.1	0.729	70.38	98.84	0.999	0.429	3.694

<LOD – Limit of Detection

Table S3. Basic validation parameters obtained for each analyte for the developed method (n, number of standards in three replicates, R2, coefficient of determination, CRM's values)

Element	n	R^2	LOD [mg/kg]	LOQ [mg/kg]	CRM (TMDA 54.6, *NCS ZC 8100 2b)	
					Certified value	Obtained value
Ag	3	0.999	0.007	0.020	12.90 ± 1.100 µg/kg	11.95 ± 0.614 µg/kg
Al		0.999	0.019	0.036	0.389 ± 0.028 mg/kg	0.389 ± 0.03 mg/kg
Ba		1.000	0.003	0.01	0.324 ± 0.019 mg/kg	0.332 ± 0.009 mg/kg
Ca		1.000	0.033	0.099	*1537 ± 68.00 µg/g	1584 ± 9.789 mg/kg
Cd		0.999	0.0003	0.001	156.0 ± 8.000 µg/kg	167.3 ± 6.559 µg/kg
Co		0.999	0.0003	0.001	305.0 ± 20.00 µg/kg	297.2 ± 4.342 µg/kg
Cr		1.000	0.001	0.003	0.421 ± 0.022 mg/kg	0.423 ± 0.028 mg/kg
Cu		1.000	0.003	0.009	0.393 ± 0.026 mg/kg	0.388 ± 0.01 mg/kg
Fe		1.000	0.024	0.071	0.367 ± 0.023 mg/kg	0.379 ± 0.006 mg/kg
K		1.000	0.027	0.082	*(14.40) µg/g	14.84 ± 1.399 µg/g
Mg		0.999	0.005	0.015	*248.0 ± 14.00 µg/g	236.9 ± 22.98 µg/g
Mn		0.999	0.002	0.004	274.0 ± 13.00 µg/kg	273.8 ± 22.08 µg/kg
Mo		0.999	0.007	0.021	292.0 ± 20.00 µg/kg	273.1 ± 10.03 µg/kg
Na		1.000	0.051	0.153	*445.0 ± 40.00 µg/g	443.5 ± 8.354 µg/g
Ni		0.999	0.002	0.006	323.0 ± 19.00 µg/kg	295.2 ± 21.68 µg/kg
P		1.000	0.003	0.009	*174.0 ± 43.00 µg/g	162.7 ± 8.129 µg/g
Pb		0.999	0.001	0.003	490.0 ± 30.00 µg/kg	460.2 ± 9.647 µg/kg
S		0.999	0.029	0.087	*(4.620) %	4.466 ± 0.01 %
Sb		0.999	0.005	0.015	*120.0 ± 20.00 ng/g	121.93 ± 1.726 ng/g
Sn		0.999	0.003	0.009	45.30 ± 3.100 µg/kg	46.14 ± 4.462 µg/kg
Sr		1.000	0.005	0.015	0.573 ± 0.031 mg/kg	0.512 ± 0.013 mg/kg
Ti		1.000	0.005	0.015	0.032 ± 0.003 mg/kg	0.033 ± 0.002 mg/kg
V		1.000	0.002	0.006	(0.089) µg/kg	0.103 ± 0.001 µg/kg
Zn		0.998	0.012	0.036	0.540 ± 0.035 mg/kg	0.566 + 0.039 mg/kg
* value for NCS ZC 8100 2b; **µg/kg; (…) – reference only						

Table S4. Pearson correlation matrix for all analyzed elements in honey and adulterated samples

Variable	Al	Ca	Co	Cu	Fe	K	Mg	Mn	Na	Ni	P	S	Sr	Ti	Zn
**Al**	1.000000	-0.393597	-0.406531	0.627649	-0.379233	-0.294455	0.777445	0.379841	-0.406150	-0.116761	0.670042	-0.356524	-0.400765	-0.411402	-0.306956
**Ca**	-0.393597	1.000000	0.969581	0.245655	0.984742	0.972654	0.090476	0.340112	0.992747	0.903541	0.226314	0.980489	0.995488	0.954025	0.877577
**Co**	-0.406531	0.969581	1.000000	0.252326	0.962456	0.974160	0.081891	0.343817	0.982554	0.921516	0.208035	0.981797	0.975998	0.982766	0.864237
**Cu**	0.627649	0.245655	0.252326	1.000000	0.268863	0.372908	0.931024	0.770212	0.246540	0.521825	0.930705	0.314973	0.252430	0.253309	0.356133
**Fe**	-0.379233	0.984742	0.962456	0.268863	1.000000	0.960628	0.104080	0.326831	0.979558	0.896181	0.232193	0.967933	0.982181	0.948939	0.871068
**K**	-0.294455	0.972654	0.974160	0.372908	0.960628	1.000000	0.220597	0.492419	0.985189	0.953511	0.344086	0.995584	0.983436	0.957898	0.880903
**Mg**	0.777445	0.090476	0.081891	0.931024	0.104080	0.220597	1.000000	0.746177	0.083439	0.389253	0.973208	0.153668	0.091407	0.081761	0.151492
**Mn**	0.379841	0.340112	0.343817	0.770212	0.326831	0.492419	0.746177	1.000000	0.379568	0.540923	0.766664	0.440800	0.375330	0.337145	0.410056
**Na**	-0.406150	0.992747	0.982554	0.246540	0.979558	0.985189	0.083439	0.379568	1.000000	0.914515	0.211751	0.992163	0.998247	0.965082	0.877461
**Ni**	-0.116761	0.903541	0.921516	0.521825	0.896181	0.953511	0.389253	0.540923	0.914515	1.000000	0.495517	0.940115	0.914702	0.897531	0.855485
**P**	0.670042	0.226314	0.208035	0.930705	0.232193	0.344086	0.973208	0.766664	0.211751	0.495517	1.000000	0.280557	0.220510	0.209099	0.307055
**S**	-0.356524	0.980489	0.981797	0.314973	0.967933	0.995584	0.153668	0.440800	0.992163	0.940115	0.280557	1.000000	0.989951	0.965601	0.883694
**Sr**	-0.400765	0.995488	0.975998	0.252430	0.982181	0.983436	0.091407	0.375330	0.998247	0.914702	0.220510	0.989951	1.000000	0.958594	0.879164
**Ti**	-0.411402	0.954025	0.982766	0.253309	0.948939	0.957898	0.081761	0.337145	0.965082	0.897531	0.209099	0.965601	0.958594	1.000000	0.863259
**Zn**	-0.306956	0.877577	0.864237	0.356133	0.871068	0.880903	0.151492	0.410056	0.877461	0.855485	0.307055	0.883694	0.879164	0.863259	1.000000

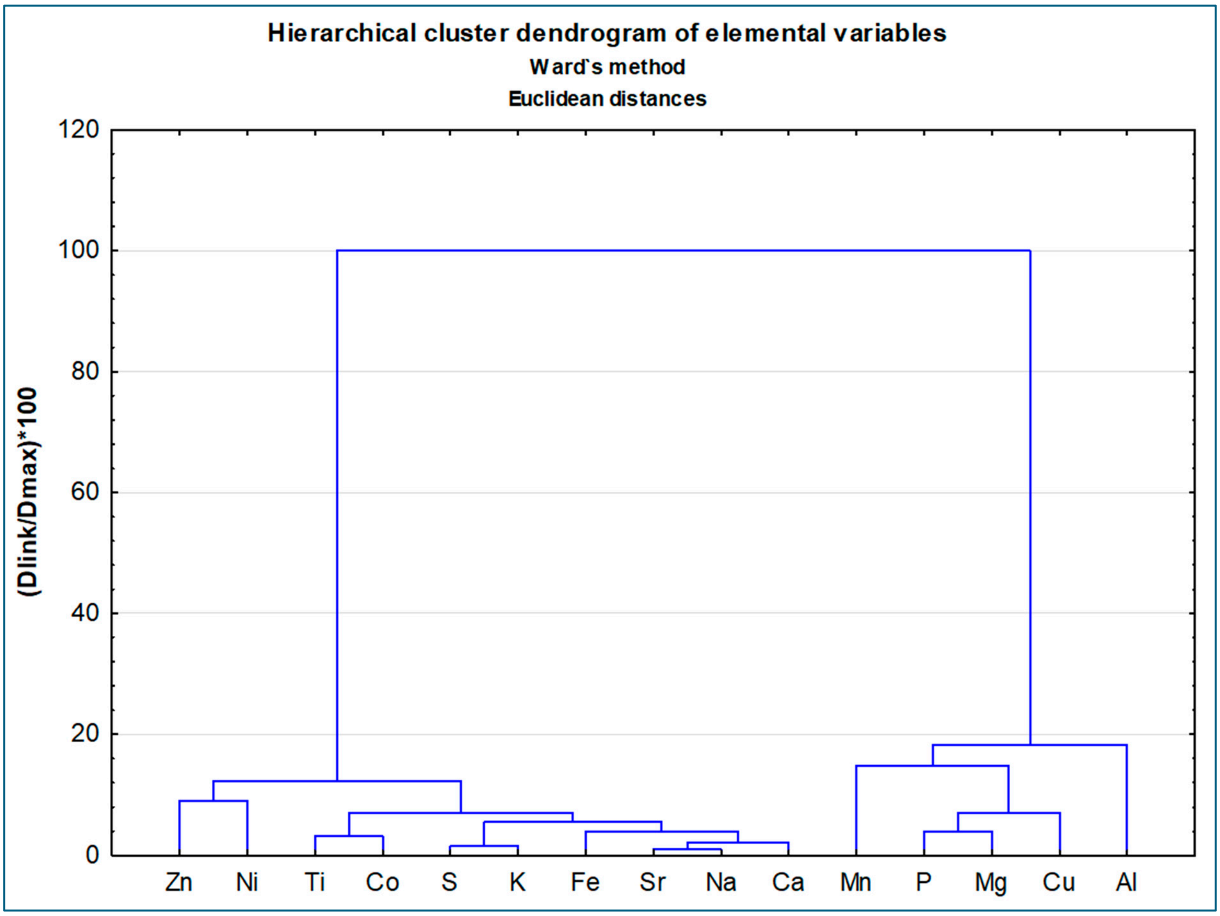

Figure S1. Hierarchical cluster dendrogram of the analyzed elements obtained using Ward's method and Euclidean distance, illustrating the similarity structure among elemental variables in all honey samples.

Table S5. Results of post-hoc Dunn's test for selected physicochemical parameters and elemental ratios

**Post-hoc Dunn test results for selected parameters p<0.05)**			
Parametr	Comparison	p-value	Z
Sugar Content	Honey vs Adulterant	0.000452	3.789718
	Honey vs Honey + Adulterant	0.002945	3.295724
	Adulterant vs Honey + Adulterant	0.185818	1.866729
Water Content	Honey vs Adulterant	0.000438	3.797543
	Honey vs Honey + Adulterant	0.004898	3.150026
	Adulterant vs Honey + Adulterant	0.133762	2.008527
K/Na	Honey vs Adulterant	0.000420	3.807976
	Honey vs Honey + Adulterant	0.011356	2.895516
	Adulterant vs Honey + Adulterant	0.072950	2.252086
Na	Honey vs Adulterant	0.070737	2.263920
	Honey vs Honey + Adulterant	0.045664	2.427070
	Adulterant vs Honey + Adulterant	1.000000	0.700649
